# Digitising legacy zoological taxonomic literature: Processes, products and using the output

**DOI:** 10.3897/zookeys.550.9702

**Published:** 2016-01-07

**Authors:** Christopher H. C. Lyal

**Affiliations:** 1 Life Sciences Department, The Natural History Museum, Cromwell Road, London SW7 5BD, UK

**Keywords:** XML, taxonomy, digitisation, nomenclature, legacy literature, zoology, botany

## Abstract

By digitising legacy taxonomic literature using XML mark-up the contents become accessible to other taxonomic and nomenclatural information systems. Appropriate schemas need to be interoperable with other sectorial schemas, atomise to appropriate content elements and carry appropriate metadata to, for example, enable algorithmic assessment of availability of a name under the Code. Legacy (and new) literature delivered in this fashion will become part of a global taxonomic resource from which users can extract tailored content to meet their particular needs, be they nomenclatural, taxonomic, faunistic or other.

To date, most digitisation of taxonomic literature has led to a more or less simple digital copy of a paper original – the output of the many efforts has effectively been an electronic copy of a traditional library. While this has increased accessibility of publications through internet access, the means by which many scientific papers are indexed and located is much the same as with traditional libraries. OCR and born-digital papers allow use of web search engines to locate instances of taxon names and other terms, but OCR efficiency in recognising taxonomic names is still relatively poor, people’s ability to use search engines effectively is mixed, and many papers cannot be searched directly. Instead of building digital analogues of traditional publications, we should consider what properties we require of future taxonomic information access. Ideally the content of each new digital publication should be accessible in the context of all previous published data, and the user able to retrieve nomenclatural, taxonomic and other data / information in the form required without having to scan all of the original papers and extract target content manually. This opens the door to dynamic linking of new content with extant systems: automatic population and updating of taxonomic catalogues, ZooBank and faunal lists, all descriptions of a taxon and its children instantly accessible with a single search, comparison of classifications used in different publications, and so on. A means to do this is through marking up content into XML, and the more atomised the mark-up the greater the possibilities for data retrieval and integration. Mark-up requires XML that accommodates the required content elements and is interoperable with other XML schemas, and there are now several written to do this, particularly TaxPub, taxonX and taXMLit, the last of these being the most atomised. We now need to automate this process as far as possible. Manual and automatic data and information retrieval is demonstrated by projects such as INOTAXA and Plazi. As we move to creating and using taxonomic products through the power of the internet, we need to ensure the output, while satisfying in its production the requirements of the Code, is fit for purpose in the future.

## Introduction

The primary source of taxonomic and nomenclatural information is taxonomic literature. Despite the growing number of databases the original published source is authoritative and provides datable interpretable content linked axiomatically to known taxon concepts. A challenge for today is how to extract the content of the last few hundred years’ publications and make it accessible in the same way that at least some novel taxonomic and nomenclatural statements are accessible (see also Pilsk et al. 2015). One means of addressing this is establishing how the taxonomic and nomenclatural acts proposed in legacy literature might be most effectively extracted and linked to (or, even more effectively, incorporated into) modern databases. The accelerating digitization of legacy literature provides an opportunity to optimise access to taxonomic and nomenclatural information. This paper focusses on zoological taxonomy, but the same arguments (with some different actors, such as MycoBank, IPNI, Index Fungorum and the appropriate Codes of Nomenclature) apply to other taxonomic domains.

The availability of both legacy literature and new publications as digital items has many advantages over solely paper format, including simple portability – it is quite possible to carry the entire corpus of publications on a taxonomic group on a single hard drive. It also provides easy availability on the internet (Pyle 2016); digitisation by large-scale initiatives such as Biodiversity Heritage Library (BHL) (http://www.biodiversitylibrary.org/), AnimalBase (http://www.animalbase.org/), the Bibliography of New Zealand Terrestrial Invertebrates-Online (BUGZ) (http://www.bugz.org.nz/WebForms/about.aspx) and Google has led to a massive increase in accessibility of otherwise difficult-to-obtain literature around the world. Ideally the process will accelerate and become increasingly efficient, with a long-term goal of creating an online digital version of all relevant literature. However, to date most digitisation of taxonomic literature has led to a more or less simple digital copy of a paper original – the output of the many efforts has effectively been an electronic copy of a traditional library. While this has increased accessibility of publications through internet access, the means by which many scientific papers are indexed and located is much the same as with traditional libraries.

While digitisation of literature is on the face of it a simple concept, it is driven by multiple objectives. These include improving security of paper copies by providing digital surrogates, improving and increasing access to publications including rare and old items, improving searchability of content by providing machine-readable versions, pursuing institutional or individual open-access policies, facilitating sharing or selling of reprints, commercial benefit, and providing a flexible publication medium. The objective of the individual or initiative responsible for the digitisation may dictate the methods used, any mark-up system employed, and the metadata provided with the digital object. This in turn may determine the uses to which the digital object may be put; for example, King and Morse (2013) show that XML mark-up employed for one use may hinder use in another area of work, looking particularly at a contrast between taxonomists who use mark-up to exploit the documents’ contents (that are relevant to them) and computer scientists who wish to explore the documents for multiple uses. In one example they noted that the scope of a marked-up text (and the editorial decisions taken by the digitisers) led to only species from Central America being tagged by the schema elements for taxonomic names; species names in the resource from other geographic regions were not tagged, and therefore undiscoverable by text mining tools created for discovery of names.

The potential of using XML-markup is being exploited increasingly in new publications, throughout the workflow of manuscript preparation to publication (Blagoderov et al. 2010). XML-markup enables content to be both displayed effectively and repurposed through delivery to relevant datasets (Penev et al. 2010). The potential for exploring that potential for legacy literature, only available as printed or OCRed text, displaying it in a way that allows users to search on content types rather than simply key terms, and access and download relevant data rather than have to manually extract it from blocks of text, has also been demonstrated by the Plazi (http://plazi.org/) and INOTAXA (http://www.inotaxa.org/jsp/index.jsp) projects (Agosti et al. 2007; Weitzman and Lyal 2006, 2015; Lyal and Weitzman 2008).

In addition to the current approaches to digitisation, and to enable us to build on the huge amount of work already done, we should consider what properties are required of future taxonomic information access systems and then develop them. Ideally the content of each new digital publication should be accessible in the context of all previous published data, and the user able to retrieve nomenclatural, taxonomic and other data or information in the form required without having to review all of the original papers and extract target content manually. Rather than building forward from the past we should be building back from the future, and bring together the many efforts across digital taxonomic information to support this. Focussing as far as possible on taxonomic and nomenclatural content, this paper will consider what such a system should deliver, and what its properties might be.

## Building the System

### Requirements

There are two general workflows and sets of components to be considered, although these are strongly interlinked. Firstly, there is the workflow necessary to discover relevant content within the digitised literature, and populate a taxonomic information retrieval system with extraction from or links to the legacy literature and other relevant resources. Secondly, there is the workflow that will operate for a user seeking information among the available databases and resources on the internet. To a large extent the requirements of the latter system will dictate the operation of the first. A third workflow should be mentioned, the production of new literature with an appropriate XML mark-up so that the contents are directly accessible together with the contents of legacy literature.

Broad requirements of the first system include:

- Generation of accurate re-purposable information from the legacy literature source;
- Generation of this information only once, not multiple times;
- Automatic population and updating of taxonomic catalogues, ZooBank, species lists, Encyclopedia of Life (EoL), etc. from digitized literature;

And of the second include:

- Accessibility of all relevant information from any (connected) source;
- Accessibility of new digital content in the context of all previous published data.

Questions that users of the system should be able to find answers to include:

- What is the correct name for an entity sought?
- What is the nomenclatural history of that name? Is it available under the relevant Code?
- What descriptions of the taxon are there?

While these are by no means all the questions that one might ask, the scope of this paper is largely limited to taxonomic and nomenclatural content. The arguments presented would, however, apply equally to components of non-taxonomic publications (physiology, immunology, ecology, biology, distribution, etc.).

In order to deliver a system that can respond to these questions, the components of that system and how they might be linked together must first be understood. Having developed the concept of the system it must be built and populated, and the content must be updated continuously and kept available to users so that current needs are met as far as possible.

The requirements of a system for this area in terms of detailed content retrievability were determined in part by a user-needs survey (Parr and Lyal 2007).

### Auto-population of sectorial systems

One of the requirements listed above is the facility to populate databases and other systems with appropriate content from the digitised literature. This needs a little further discussion, since it dictates how digitisation should be performed and elements within the digital content should be tagged. Examples of this already exist for digitised legacy literature and new publications. Both INOTAXA and Plazi expose their content to EoL, and Plazi further supplies data to ZooBank. The journal *ZooKeys* supplies data to GBIF, ZooBank, EoL and Species-ID, and the sister publication for open access biodiversity data, *Biodiversity Data Journal*, implements for the first time a pre-submission mark-up of various types of highly atomised biodiversity data. This allows for automated export of Darwin Core Archives of treatments, occurrences etc. and their direct indexing by GBIF and EOL (http://biodiversitydatajournal.com/about#Globallyuniqueinnovations). However, rather than simply deliver names and the associated citations to ZooBank, for example, much more is possible with appropriate data and metadata capture from the publication. Requirements for the availability of new names under the ICZN are set out in the Code itself (an abbreviated list is presented in Table 1). If these requirements could be turned into a set of standard queries, and the schema built to enable the appropriate data and metadata from the publication to be delivered, it would open the door to automatic assessment of nomenclatural availability through algorithms built into ZooBank, and populate ZooBank with not just the name but also the availability status with reasons for that status.

**Table 1.** Some of the criteria for availability of names under the ICZN, and potential to extract information to determine availability. Note that some of these are required or appropriate only for the current amended Code (http://www.nhm.ac.uk/hosted-sites/iczn/code/), and the date of publication of the original text will dictate what criteria are applicable.

Criterion for availability	Requirement
Publication is obtainable in numerous identical copies	metadata
Publication: If non-paper, produced by a method that assures widely accessible electronic copies with fixed content and layout, and registered in ZooBank.	metadata
Publication not excluded by Article 9	metadata
The name is published using the Latin Alphabet	metadata
For species-group names, name agrees in gender with the genus name	markup + algorithm
For family-group names, name has a permitted ending	markup + list
For family-group names, name has suffix appropriate for rank given	markup + list + algorithm
For family-group names, name is based on the genus name stated	markup + algorithm
Name not already registered	markup + ZooBank search
Name contains more than one letter	markup + algorithm
Genus in which new species-group name is placed (if applicable)	markup
Name not published as a synonym but as a valid name	markup
Valid genus name on which new family-group name is based	markup
Type species of new genus-group name (including original combination, author and date)	markup
Description of taxon, or bibliographic reference to a description, is part of publication	markup + algorithm

This clarifies a requirement that when legacy literature is taken from a non-digital to a digital state, not only should the original be viewable and searchable, but there should be the potential for subsets to be viewed, extracted on request, and be able to be analysed separately. Extending this beyond the elements related to taxonomy and nomenclature, the ability to extract components in order to be able to work with these independently opens the door to those components being repurposed in different applied contexts (e.g. taxa grouped according to where they have been recorded geographically or with what other taxa they were collected, by classification etc.). This also, of course, opens the door to relevant content being extracted (ideally automatically) to populate and enhance sector-critical systems (e.g. ZooBank, GBIF). This can be done through appropriate mark-up in XML.

A first step to this increased functionality is the user-needs assessment mentioned above. While general requirements are fairly straightforward to assess, there are differences in perception of priorities and significant needs, and consequently how the texts need to be marked-up to enable these to be met. One distinction that is already clear is whether mark-up should focus on taxon treatments (the policy of Plazi) or the complete content (the policy of INOTAXA). Given that relevant content can be found outside taxon treatments in the corpus of taxonomic literature, for a full system satisfying the requirements above it appears that full-text mark-up is required. This is discussed further in section 2.2.4 below. Analysis of different XML mark-up systems by Penev et al. (2011) revealed that the technology was only part of the issue, and that editorial policies of the groups managing the systems were also significant. These include the sections of the texts to be marked up, and also the level of interpretation of ambiguous content and how such interpretation is exposed to the user. Such editorial policies should also be harmonised, at least to a set of agreed practices or practice alternatives.

### The functional components

**Figure 1.**
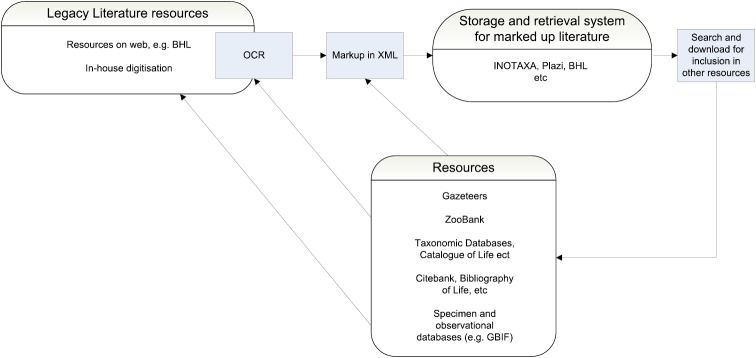
Outline workflow to acquire, put into a suitable format, retrieve and utilize legacy literature.

The basic workflow of a system to acquire, put into a suitable format, retrieve and utilize legacy literature is given in Fig. 1. The system is self-reinforcing; the more populated the vocabularies and glossaries generated through extraction of the digitised elements become, the stronger they will be as a tool to assist the automated or semi-automated OCR and mark-up processes.

Many components of the system already exist. These are the initiatives, organisations and individuals digitising and hosting taxonomic literature, databases of names and other taxonomic and nomenclatural information, museum databases and the aggregators such as GBIF that make specimen data widely available and are developing interoperable systems, and people working on standards, particularly with Biodiversity Standards (TDWG). Some of these are already communicating more or less effectively; others less so or not at all.

Patterson et al. (2010) set out a vision of access to the varied and massive amount of biological information on the internet through using taxonomic names to index content, and in particular the Global Names Architecture (http://www.globalnames.org/) (GNA) (see also Pyle 2016). A core of the GNA is the Global Names Usage Bank (GNUB) which is intended to index all published statements about life on Earth. They identify a first iteration of this as ZooBank (http://zoobank.org), the registry for names of animals developed by the International Commission on Zoological Nomenclature (Pyle and Michel 2008, 2009). They also refer to the Global Names Index (http://gni.globalnames.org/) (GNI), which is a simple index of all unique name strings (whether correctly or incorrectly spelled), with or without author attributions. Other actors (at a generic level) identified include Biodiversity Library’s CiteBank (http://citebank.org/), containing citations for biodiversity publications, and taxonomic catalogues of species. Page (2013) uses taxon names to link a range of different taxonomic products and resources to texts at the article level, using a system ‘BioNames’ (http://bionames.org) that combines classifications from GBIF and GenBank, images from EoL, animal names from the Index of Organism Names (ION) and bibliographic data from sources including BHL and CrossRef. Many of these actors are built up by collaboration among many contributors; GBIF names are derived from nearly 15,000 datasets of observations and specimen records, plus the names from the Catalogue of Life (CoL) (http://www.catalogueoflife.org/); the latter is compiled from 143 contributing databases.

Records of taxonomic names can be considered in three classes: *name lists*, containing name strings, with no taxonomic or nomenclatural information (such as stored by the GNI); *nomenclators* such as ZooBank, which are lists of names with nomenclatural information but no taxonomic interpretation (i.e., will include all names as ‘equal’, not distinguishing between junior and senior synonyms, for example); and *catalogues and checklists*, which are lists of names that include taxonomic information such as synonymies, validity and current combinations. What is perhaps surprising is that there is no clear agreement of what data are collected where or, indeed, how they should be stored in a database. Taxonomic databases and catalogues are the least consistent in content. They will almost always contain the valid name as understood by the compiler, although whether this has been checked either against the most recent expert taxonomic treatment or consensus, or for compliance with the ICZN, is rarely stated. Such databases may contain valid names and combinations, obsolete combinations, original combination and orthography, obsolete ranks and systematic positions, junior synonyms, subgenera, misspellings, type taxa and full citations for each – or they may not. ZooBank includes only nomenclatural acts as covered by the Code (mostly ‘original descriptions’ of new scientific names for animals, but other acts may include emendations, lectotypifications, and other acts as governed by the ICZN Code), publications that contain nomenclatural acts, authors of those publications, and type specimens for scientific names of animals (the last is considered provisional and is not yet fully implemented in ZooBank). The data quality is generally good, reaching excellent where the entries have been checked. The names accessible through the GNI are just that – names, with no qualitative information. GBIF is similar to the GNI in part of its name provision; it serves names from taxonomic databases contributing to the Catalogue of Life but also names attached to specimens and observations that may or may not be reconciled to those for CoL.

### Digitised legacy taxonomic literature

There is a large quantity of digitised content on the web. At the time of writing, the BHL made more than 45.4 million pages (from 156,114 volumes – 91,167 titles) freely available. Another resource, BUGZ, contains a literature database of more than 16,000 articles and full-text indexing of more than 200,000 pages. These are not the only sources. Therein, oddly, lies a problem. There is a very large number of both large and small-scale libraries, ranging from these major initiatives to individual researchers’ web-sites. Consequently, it can be problematic for a human user to find a required article, and the user may need specialised understanding of the content and priorities of different libraries, including where to find particular collections. This issue is at least partially addressed by the ReFindIt tool (http://refindit.org/index.html), an integral part of Bibliography of Life (http://biblife.org/). ReFindIt searches across multiple bibliographies such as CrossRef, Mendeley, GNUB, Refbank, DataCite and DOIs. Some articles can be found directly through web browser searches, but this is not the case for the contents of some of the larger initiatives, which are only findable using the search systems of the initiatives themselves. These search systems can be restrictive, and may only allow searches using pre-determined criteria requiring information not necessarily available to all users (such as the full name string of the journal in only one form – a problem given the uncertainty often associated with journal names and their abbreviations – see Pilsk et al. 2015). Another issue with some initiatives is that digitisation has been carried out, for very good reason, at volume level, and consequently the metadata are at this and not the article level. This problem is compounded by the difficulty of automatically identifying the beginning and end of articles during digital capture or subsequent parsing. There is thus a mismatch between the metadata served and the search unit often employed by the user, who is often looking for articles or even taxon treatments.

There are various possible solutions to the problems outlined above. BHL, for example, has a browse function using a number of elements (titles, authors, subjects, origin of publication (using a map), publication year, language, contributors and collections). Another effective means would be to expose all digital content to direct search by internet browsers; Google books uses this method, as do many smaller collections. However, while this would improve current accessibility, it does not necessarily provide easy access within a workflow since, unless the search criteria are very rigorous, sorting through multiple irrelevant search results may mean the user abandons the process before finding a required resource. In addition, links between related papers (separate parts of the same paper published at different times, supplementary works, etc.) are generally not emplaced in the digital resources (Page 2016).

A second step is to facilitate machine-location of digitised texts. This might require the adoption of standard search routines that could operate as a web service across different sites. It entails adding metadata to digitised objects that would enable searches for units or entities that are of particular value to the expected users. Such units, for taxonomists, will include article-level publications (articles, chapters) and text elements within articles (for example taxon treatments). It will also require increased use of unique identifiers such as DOIs (Page 2016).

For inclusion in a workflow from digitisation to an interoperable system, large literature resources such as BHL have major advantages in addition to the very large content (and thus the easier location by users setting their own priorities). These include the standardisation of OCR that has taken place, the facility to download in one of a number of formats easily, and the persistence of the source.

A relevant although non-technical issue is copyright, which prevents open access to many publications. This has been discussed recently by Agosti and Egloff (2009) and Patterson et al. (2014). The former authors, considering taxonomic descriptions, opined that these are in the public domain and can be used for scientific research without restriction, whether or not contained in copyrighted publications. Patterson et al. (2014) focussed on scientific names in catalogues and checklists, and concluded, similarly, that these are not creative under at least US and EU copyright law and could be freely shared, although they noted the importance of providing credit for the original compilers.

### Optical Character Recognition and accuracy of the digitised content

The majority of digitised legacy literature on the web is provided as an image of the page plus an underlying text generated by Optical Character Recognition (OCR), so that the software can find words or phrases within the text in response to a search. In some cases there is no OCR, while other sources provide only the OCR. While a scanned document may appear accurate as an image, an underlying (or alternative) OCRed text may not fully correspond. For this reason a search of a PDF may not retrieve all instances of a term, even when the term can be seen plainly on the screen, an issue compounded by non-Roman alphabets not properly OCRed in that language.

OCR techniques are not as effective for taxonomic literature as one might wish. There are particular problems associated with using OCR on digitised legacy literature, where character recognition may be compromised by factors including old fonts, foxing of the pages, transfer of print or image elements between pages, and translucency of paper so the image displays text from the obverse and converse of the page. However, while OCR can, subject to resolution of these problems, recognise most terms, the effectiveness of many programmes in recognising technical terms, taxonomic names and geographic place names is relatively poor. Consequently searches on such terms, even within specialist sites like BHL, do not reveal the full content. These problems have been addressed by, among others, Morse et al. (2009) and Akella et al. (2012); the systems developed need to be embedded effectively in functioning workflows, and currently for the majority of digitised and OCRed legacy literature the issues still remain.

To assist OCR software to recognise non-standard terms, specialised glossaries and vocabularies would be of value, although use of contextual information and machine learning are also both important. For example, a list of Parties for the names of authors, editors, collectors etc. (including the variations in the possible strings involved resulting from use of initials or full names, order of initials etc.) would facilitate recognition of more complex names. Other glossaries that would assist in text recognition are geographical place names, technical terms and of course taxonomic names; these, in addition to facilitating accurate OCR, would, through their distribution in the text, facilitate recognition of different text elements: a paragraph with many geographical place names is likely to be either a paragraph outlining distribution, or a list of specimen localities. A list of journal names and abbreviations would assist in good OCR of bibliographic elements (since references are often in a variety of languages, which can also pose a problem for OCR). Such a list would have to be open, since abbreviations of journal names are far from standard in zoology; it might form a part of a master bibliography as discussed below. As indicated above, such glossaries and vocabularies can be strengthened by the products of OCR and mark-up.

Even with the assistance of tools such as glossaries and technical vocabularies, text may contain terms of use for searches but which OCR software cannot simply recognise, such as abbreviations of author names and generic names, and these may need manual interpretation. This is also an issue for parsing text into XML, as discussed below.

### Marking up the content

In order to transform OCRed content into a resource that can be searched effectively for classes of content (e.g. names, places, taxonomic acts etc.), it needs to be marked up into XML or other system that permits computer searching. This paper is not the place to address whether XML or RDF is the most appropriate system for development (Penev et al. 2011). Instead, it deals with the currently-developing systems, which are largely based on XML. The simple requirements are that mark-up accommodates the required content elements and is interoperable with other XML schemas relevant to the topic so that relevant content can be accessed wherever needed. Weitzman and Lyal (2007) attempted to map the various potentially interacting standards in play for taxonomic content, indicating where interoperability is required. Such interoperability might be achieved either by appropriate mapping between elements used in different schemas, or by re-use of schema components. Both positions have positive and negative aspects, and both approaches are in use. There are several schemas and DTDs written to manage taxonomic literature, particularly TaxPub, taxonX and taXMLit, the last of these being the most atomised. These have been compared by Penev et al. (2011).

Different schemas provide different levels of atomisation of the content. The greater the atomisation of the mark-up, the greater are the possibilities for data retrieval and integration, although this can carry a cost burden, since the greater the atomisation the greater the effort needed to parse content into the schema. However, this must be driven by the long-term goals and requirements. Some of the elements required have been identified in Table 1 – those providing sufficient data and metadata to automatically assess nomenclatural availability of a name. These elements would have to be separately tagged. Other elements are those which users would typically wish to search for, as outlined in the Introduction, and include both publications and subsets of publications. Within a publication the ‘top level’ is article-level (including chapters), these necessarily including the full publication metadata such as date of publication (both cited date and ‘true’ date, the second of which may require annotation) and other metadata that will enable a user (either human or automated) to assess its publication status and some of the availability criteria under the ICZN.

An important subset of publications at the article level, and that to which most attention has been paid to date, is the taxon treatment, since this is a text element of particular interest to taxonomist target users (Weitzman and Lyal 2004; Kirkup et al. 2005; Agosti et al. 2007; Sautter et al. 2007; Lyal and Weitzman 2008; Penev et al. 2011). For a system to have the most effective functionality, a search should retrieve all treatments of a taxon and, in some circumstances, its hierarchical children (subspecies, infrasubspecies, synonyms); this of course requires all of these treatments to have been digitised and marked up, and there is an issue of cost-effectiveness that will dictate to what extent treatments are digitised and to what extent they are captured in databases. For taxonomic and nomenclatural purposes the digitisation of a treatment should include in its identified sub-elements all components that will support identification of nomenclatural availability and validity under the ICZN.

Within articles the automated recognition of taxon treatments for mark-up is reliant on text and formatting recognition. Curry and Connor (2008) identified several standard text elements within treatments: name, synonyms, diagnosis, description, distribution, material examined and discussion. Unfortunately such text elements are neither uniform in formatting nor universal in their presence, so different publications may need different ‘rules’ to allow their recognition. The largest resource offering publications broken down into taxon treatments, Plazi, relies on manual intervention to recognise beginning and ends of treatments. Progress is required on use of OCR systems that retain text formatting, coupled with a natural language programming approach and perhaps use of catalogue resources to improve recognition of both article and treatment boundaries. Less work has been done on recognition of other large text elements such as bibliographies or checklists, but the priorities for these would be usefully developed and mechanisms to recognise them explored.

Other subsets of taxonomic publications that might be required might include taxon hierarchy (which may be an implicit construct from a publication rather than and explicit section within that publication), bibliography, diagnosis and description, biological associations, specimen data and character statements, taxon citation ([name] [author] [date] [nomenclatural/taxonomic act]), the original description citation ([name][author][date][reference]), subsequent taxonomic or nomenclatural changes citations, nomenclatural, taxonomic and other data or information.

Some of these subsets are more strongly structured data than cursive text, such as citations, bibliographic records and specimen data. In such cases the content, once marked up and made available through a suitable interface including both human searchability and web services, should be downloadable and repurposable, and consequently delivered or extractable in a common standardised format (e.g. Darwin Core for the specimen data). BHL already does this with the full content of publications, making the text available not only as HTML but also as downloadable PDF, OCRed text etc. INOTAXA makes specimen data extracted from text available as a spreadsheet and exposes taxon treatments and subsets to harvest by EoL, while Plazi extracts specimen data in Darwin Core and supplies it to GBIF. A concomitant to this is that content that is not required by the user (e.g. the text around specimen data) should be capable of exclusion from the retrieved content, and ideally the user should be able to retrieve such data without manually reviewing the whole paper and retrieving the required data by copying text items. This serves to define elements that need to be recognised in the schema.

A further requirement for successful mark-up is the population of implicit terms. For example, specimen data listed in papers is often incomplete for many of the specimens; the country may only be provided for the first of multiple localities within that country, or specimens with similar data to others may simply be listed “as previous, but dates …”. If the specimen data are to be downloadable the country and all other data must be available for all the localities not just the first. Similarly, terms such as ‘loc cit’ and ‘ibid’ must be replaced as automatically as possible with the full citation.

Despite the work done in automating mark-up to date (Sautter et al. 2007a; Penev et al. 2011), the process is unlikely ever to be fully automated. Experience has shown that texts can hold many ambiguities that require interpretation, and for which automation is unlikely to be either cost-effective or successful, although semi-automation is certainly possible and has been used both in the Plazi and INOTAXA projects. Future work should focus on increasing the proportion of automation and reducing human input. This requires the process to accommodate the workflows of those most likely to be able to resolve ambiguities, who are likely to be taxonomic experts. That said, progress has been made with using crowd-sourcing to mark-up legacy documents (Thomer et al. 2012), and a system ultimately may make use of automation, crowd-sourcing and expert review and annotation.

A required resource for a mark-up workflow is a location where marked-up texts can be stored. This may have to incorporate several versions of a text, given that mark-up may not be completed for a text, and may even be done by more than one person. This repository may be the same initiative where the original document was sourced, or could be another place. However, ideally it should be fully accessible for users, including whatever search system is put in place for accessing content according to user needs. This last point implies a single gateway for searches, which could either search locally or, more effectively, across sites (where marked up text in appropriate formats is also exposed to searching). The search system itself will need to be in line with the requirements outlined above and capable of refinement as other user demands develop. Two systems have been developed in recent years that satisfy at least some of the needs, the Plazi and INOTAXA systems. Both work with a single repository of documents marked up within their projects to particular standards.

### The global bibliography

Locating texts, whether marked up or not, ideally will involve indexing in some manner, and this may be a function of a global bibliography. This has already been mentioned above in the context of assisting mark-up through identifying beginning and end of articles, but also has a function here. The bibliography will have to include standards for citation of both library and taxonomic sectors, which do seem to differ, the latter including abbreviations and contractions understood (and used) by the sector but not appearing in library catalogues. Many journals, for example, have ‘standard’ abbreviated formats required by some publishers for use in bibliographies, and some taxonomist names are similarly abbreviated in a standard fashion [e.g. ‘L.’ for Carolus Linnaeus (= Linnaeus, C. = Linnaeus, = C. Linnaeus, = Carl Linnaeus, = Carl Nilsson Linnaeus = Carl von Linné = Carolus a Linné, etc.) (but not for his son)]. A vocabulary of abbreviations and alternate name strings for the same entity, accompanied by suitable unique identifiers, will enable some mark-up issues to be resolved automatically. There will still be a requirement for de-duplication and interpretation, although the global bibliography itself will assist in resolving them, especially if combined with the Global Names Architecture. For example, the microcitation ‘Smith 1995’ is undoubtedly ambiguous, but if combined with a taxonomic name (‘Aus bus’ Smith 1995’) which can be found in a taxonomic database (using, perhaps, the Global Names Architecture) and then linked through that to a bibliographic reference, the microcitation (and author) can be resolved to known entities and the taxon name can be added to the metadata associated with the reference in the global bibliography.

As discussed above, automated recognition of articles within volumes is not straightforward; textual or formatting cues differ widely between publications, and many OCR conversion programmes (which allow the text to be searched) do not record original text formatting, thus stripping potential cues from the machine-readable content. Article recognition might be facilitated through access to bibliographic databases as discussed above (using web services); the title namestring might be used in conjunction with the page number to find the beginning of the article, and the final page number from the reference to find the end, for example. This requires the population of (open-access) bibliographic databases. There have been some attempts at compiling such databases, but this is a major task and needs automation where possible. Botanical literature between 1753 and 1940 has been captured by Stafleu and Cowan (1976–1988), Stafleu and Mennaga (1993–2008) and Donn and Nicholson (2008, 2008a), and is now online in database format (http://www.sil.si.edu/digitalcollections/tl-2) (Pilsk et al. 2015). This gives information not only on publications but also provides standard abbreviations of author names, which is of great value in disambiguating these. The BHL developed CiteBank (http://citebank.org/), and currently collects citation details when users choose to download a part of a digitised volume – users are permitted to download any set of pages they required from within a digitised volume; however, to do this they must enter the citation of the article being downloaded, which is then retained as metadata. Under the recent ViBRANT project the ‘Bibliography of Life’ (http://biblife.org/) was created in order to store, de-duplicate, parse, curate and share references, linked to a set of free services; this currently holds more than 215,000 references.

### Interoperability and the information network

Implicit in all of the above is interoperability. How this is achieved is largely beyond the scope of this paper, but clearly all of the different databases and repositories in any system must be able to communicate and share data. There must also be the ability for users at any point to annotate records. This is discussed at some length in the recent Global Biodiversity Informatics Outlook (http://www.biodiversityinformatics.org/). One key component that has already been alluded to is that all of the participating actors must agree to some standard elements for their schemas and databases. Taxonomic name strings must be atomised in the same way, taxonomic acts, authors, citations, specimen data and so on must all be recorded in such a way that interoperability is possible. Without this the system cannot deliver benefits.

### Sociological factors

Irrespective of technological advances, the major barriers to progress are likely to be sociological. Across the communities of scientists who might be expected to contribute to building a system there are very widely differing levels of understanding of what is needed, different skill levels in biodiversity informatics and other relevant technologies, and different levels of understandings of the current possibilities for data sharing and how these data may be used. At one end are individuals, usually highly experienced taxonomists, who still compile their information on stand-alone databases, spreadsheets or word-processed documents; at the other are computer-focussed builders of innovative bibliographic research tools, but who may have little engagement with taxonomic research. No tool is easily incorporated into a workflow unless it delivers what the users need more simply and effectively than it is delivered by familiar methods. This means that tools must be simple to use and not change too much or too rapidly. What is needed is not a set of beta-version products as generated by a succession of independent research projects but relatively stable well-documented production tools. This is not a straightforward requirement; we are all familiar with the truism that it is simpler to get a grant to develop a novel system than to obtain money to populate an existing one, and developments are happening so rapidly that it is difficult to harmonise them all. That said, BHL is an example of a component that has placed itself in multiple workflows effectively, and is growing by adding functionalities. ZooBank similarly has embedded itself in workflows, to the extent that for some journals and authors inclusion of ZooBank registration numbers is best practice, and initiatives such as NCBI (GenBank), BHL and GBIF are building it into their functionalities. It is also establishing cross-links to legacy literature – an important development which will support the building of the system discussed in this paper.

A further sociological factor is the independence of database compilers. Each database being compiled is created for a particular purpose and owned by one or more individuals. This has led to some taxa being covered by more than one taxonomic database (sometimes at global and regional levels), while others are not covered at all. In some cases the same information has been collected multiple times from primary and secondary literature. As noted above, each of these databases may be compiled using non-interoperable systems and without clarity or even consistency on the nomenclatural and taxonomic elements it contains. Assistance to use standardised systems that can connect to others to download and share information would gain more uniformity among database owners and facilitate their work. An analogous system is that of genealogical research, a very popular hobby in some parts of the world. There are many individual researchers but the tools they use are fairly standardised, many use a common (GEDCOM) format to exchange content, and many have easy links to on-line resources to find and download relevant data, and to upload content. Such programmes also exist for taxonomists, such as SpeciesFile (http://software.speciesfile.org/HomePage/Software/SoftwareHomePage.aspx) or Mantis (http://140.247.119.225/Mantis/index.htm) but as yet none can be considered as standard tools.

## Summary

Taxonomic research and its nomenclatorial supporting structure are embracing the digital environment. However, to an extent we are still treating each resource – databases, checklists, taxonomic publications, faunas and floras etc. – as separate stand-alone items. Instead we should be mainstreaming the idea of bringing all of these together in a digital environment. One means to do that is through use of XML. Both legacy and new literature can be marked-up into dedicated schemas, and the more atomised the mark-up the greater the possibilities for data retrieval and integration. Mark-up requires XML that accommodates the required content elements and is interoperable with other XML schemas, and there are now several written to do this, particularly TaxPub, taxonX and taXMLit, the last of these being the most atomised. A need now is to automate this process as far as possible. With such mark-up and display in an appropriate platform, the door is opened to dynamic linking of new content with extant systems: automatic population and updating of taxonomic catalogues, ZooBank and faunal lists, all descriptions of a taxon and its children instantly accessible with a single search, comparison of classifications used in different publications, and so on. To move to such a model will require an agreement on vision and wider acceptance of both standards and desirable properties of digitised output.
